# Variances in cellular sedimentation behavior as an effective enrichment method of hydrocarbon-overproducing *Micrococcus luteus* strains

**DOI:** 10.1186/s13068-018-1286-6

**Published:** 2018-10-20

**Authors:** Angel Angelov, Maria Übelacker, Wolfgang Liebl

**Affiliations:** Department of Microbiology, School of Life Sciences Weihenstephan, Technische Universität München, Emil-Ramann-Str 4, 85354 Freising-Weihenstephan, Germany

**Keywords:** Biofuels, Microbial hydrocarbons, Fatty acid-derived fuels, Olefins, *Micrococcus luteus*, Screening, Cell flocculation, Bacterial hydrocarbons, *Micrococcus*

## Abstract

**Background:**

Aliphatic hydrocarbons of microbial origin are highly interesting candidate biofuels because these molecules are identical or very similar to the main components of petroleum-based gasoline and diesel fuels. The high-GC Gram-positive bacterium *Micrococcus luteus* is capable of naturally synthesizing long-chain, *iso*- and *anteiso*-branched alkenes which are formed via the head-to-head condensation of fatty acid thioesters by a dedicated enzyme system. The present study describes the relation we observed between olefin production and cell buoyancy in *Micrococcus luteus* and the use of this phenotype to simply and efficiently separate cells from a mixture based on their hydrocarbon content.

**Methods:**

We generated *M. luteus* mutants producing different amounts of olefins and used them in mixing and sedimentation experiments, olefin content analysis by GC-MS and in equilibrium centrifugation in Percoll gradients.

**Results:**

We found well-detectable differences in the buoyant densities of the examined strains, which correlated with the amounts of hydrocarbons produced by the cells. We also demonstrate how our observations can be used to simply and efficiently fractionate cells based on their hydrocarbon content.

**Conclusions:**

In summary, we show that cultures of *M. luteus* cells sediment at distinct rates depending on the amounts of alkenes produced. Our results indicate that buoyant cell density is the primary cause for the observed differences in sedimentation behaviour. The simple separation strategy described here can be a valuable tool in various mutagenesis and enrichment protocols, aimed at generating and isolating strains with increased olefin productivity.

**Electronic supplementary material:**

The online version of this article (10.1186/s13068-018-1286-6) contains supplementary material, which is available to authorized users.

## Background

Biosynthesized hydrocarbons are an attractive target for microbial metabolic engineering, because these molecules can be very similar or identical to the main components of the petroleum-derived fuels and are, thus, fully compatible with the existing infrastructure [1–4]. Several bacterial pathways have been discovered in the last years which lead to different types of hydrocarbons, among them is the cyanobacterial medium-chain alkane pathway [5], or the olefin biosynthesis pathway, which was first elucidated in *Micrococcus luteus* [6] and was later found to be present in various bacterial lineages [7]. The development of future bacterial hydrocarbon production will depend on the availability of tools to perform rapid genetic engineering as well as on methods to evaluate the hydrocarbon content in the engineered strains. Classically, microbial hydrocarbons and other fatty acid-derived fuels are measured by solvent extractions of bacterial cultures followed by gas chromatography–mass spectrometry (GC–MS). Although this is the most accurate and reliable method to measure such compounds, it is not well suited for high-throughput settings, for example, in a screening of a random mutagenesis library. For high-throughput assays, the fluorescence emitted by the hydrophobic dye Nile Red has been established as a convenient proxy for the amounts of hydrocarbons and ketones in bacteria [8] and Nile Red-based screenings have been employed in the isolation of free fatty acid overproducing mutants of *Escherichia coli* [9]. Classical screening approaches, like the Nile Red screening above, rely on the isolation and probing of single colonies (or liquid cultures). The present study describes the relation we observed between cell buoyancy and olefin production in *Micrococcus luteus* and the use of this phenotype to simply and efficiently separate cells from a mixture based on their hydrocarbon content. Selective enrichment of cells from mixed populations based on their size has been demonstrated before in continuous fermentations in a specially designed fermenter [10, 11]. With the population heterogeneity in hydrocarbon-producing *M. luteus* cells described by us, it may be feasible to apply similar fermentation strategies to increase productivity.

## Results and discussion

**Fig. 1:**
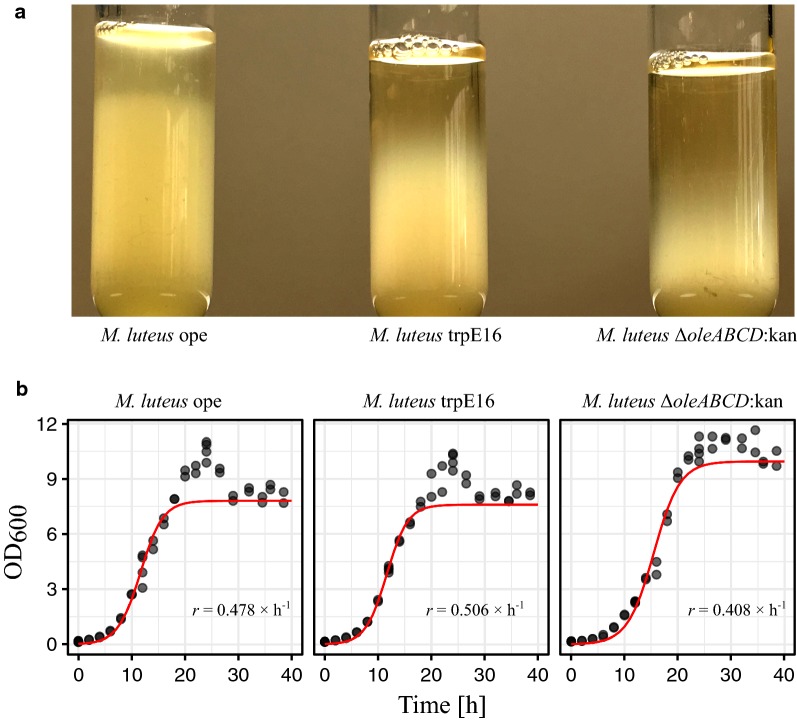
Settling of stationary phase-grown cultures (**a**) and growth curves (**b**) of *M. luteus* trpE16 (wt), the olefin-overproducing strain *M. luteus* ope and of the olefin gene cluster knockout strain *M. luteus* Δ*oleABCD*:kan. The strains were grown in rich medium (LB) for 40 h with shaking at 30 °C and afterwards left undisturbed for 4 h. The growth curve measurements were performed with 4 independent cultures, the growth rate constants (*r*) were estimated by fitting the absorbance data points (black dots) to the standard form of the logistic equation (red line), using the drc package in the R programming language [18]

**Fig. 2:**
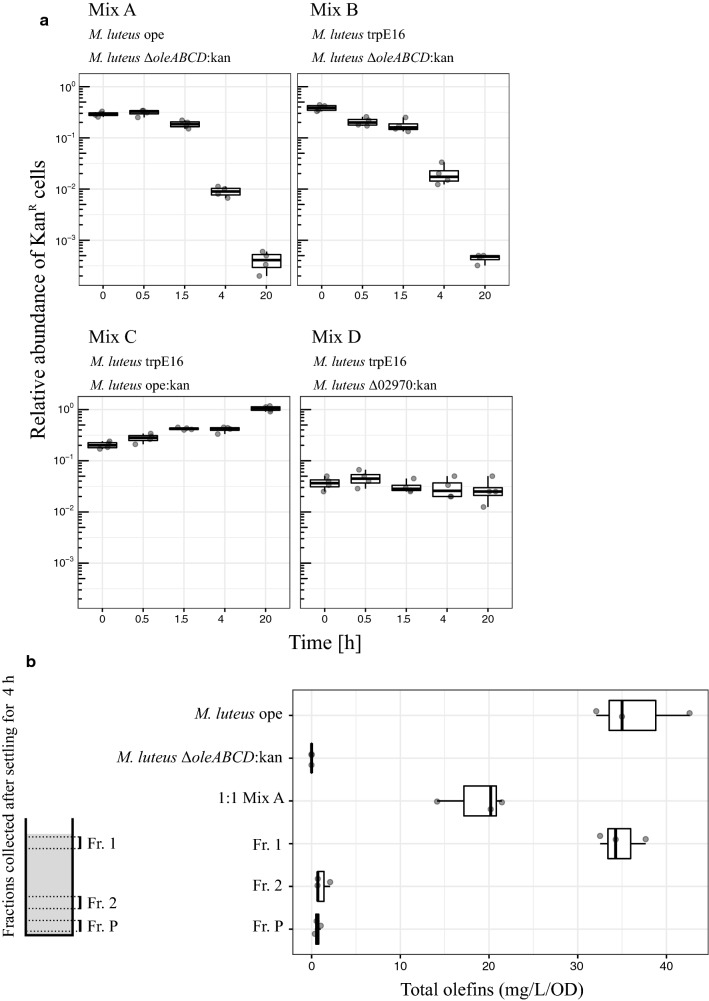
**a** Kinetics of the separation of olefin-producing and non-producing *M. luteus* cells in mixtures of two strains, measured by estimating the frequency of an associated kanamycin resistance marker. The 10-ml mixes contained 2 strains grown to the stationary phase in complex medium (48 h of growth in LB). Samples from the upper phase were collected (0.1 ml) at the indicated time points and dilutions were plated on LB plates with and without kanamycin. The LB kanamycin/LB ratio represents the relative abundance of the kanamycin resistant cells in the mix. The box plots show the data from 4 replicates (*n* = 4). **b** Total olefin content, determined by GC–MS, in fractions of Mix A (consisting of *M. luteus* ope and *M. luteus* Δ*oleABCD*:kan) after separation by sedimentation for 4 h. The cultures of the two strains were grown separately for 48 h in rich medium and 250 ml of each were mixed in a 1:1 ratio. The fractions (40 ml each) were normalized by cell density and subjected to 4 separate extractions with hexane. Each hexane extract was analyzed by GC–MS and the total olefins were quantified using an internal standard (triacontane). The boxplots represent the quantification data from four extractions (*n* = 4)

During our work on engineering the olefin biosynthetic pathway in *Micrococcus luteus*, we repeatedly observed that the cells of liquid cultures expressing different amounts of olefins show distinct sedimentation behavior. For example, when cell suspensions of stationary phase-grown *M. luteus* trpE16 (parental strain, [12]), *M. luteus* Δ*oleABCD*:kan (a non-hydrocarbon-producing mutant of trpE16) and *M. luteus* ope (an olefin-overproducing variant of trpE16) were left undisturbed for 4 h, a clear difference in the speed with which the cells sedimented could be observed (Fig. 1a). These differences were not due to different growth stages of the three strains, as they showed almost identical growth kinetics under these conditions (Fig. 1b). Also, there were no noteworthy differences in the microscopic appearance of the cells, i.e., regarding cell size and cell aggregation behavior (see below). We then asked if this settling phenotype can be sufficient to separate hydrocarbon-producing from non-producing cells in a mixed population simply by collecting the upper phase of a cell suspension after letting it to stand undisturbed for several hours. For this, cell suspensions of a mix of two different *M. luteus* strains with distinct olefin content were prepared, where the mixes contained approximately equal amounts of the two strains. In these cell mixes, one of the strains carried a chromosomally integrated kanamycin resistance marker, which permitted us to easily determine the ratio of the strains in the samples by plating on agar plates with and without antibiotic. From a glass tube with a 10-ml cell suspension mix left standing unmoved at 20 °C, 0.1 ml of samples from near (about 5 mm) the top was collected at different time points and dilutions of the samples were plated on LB plates with and without kanamycin to determine the relative abundance of the kanamycin-resistant cells. When olefin-non-producing cells were mixed with either the wild type or the *M. luteus* ope strain, the fraction of olefin-non-producing cells in the upper phase decreased dramatically, leading to a more than 1000-fold enrichment of olefin-producing cells in the upper phase after 20 h (mixes A and B in Fig. 2a). Control experiments, performed with mixtures of the wild type and an olefin-overproducing strain carrying a kanamycin marker (trpE16 and ope:kan, mix C in Fig. 2a), showed that this separation was not affected by the antibiotic resistance gene. Another control involved mixing of the wild type and a strain with a kanamycin insertion not affecting the olefin production (trpE16 and Δ02970:kan, the latter being a deletion strain for a putative transcription regulator, mix D in Fig. 2a), where the strains ratio was deliberately set at ~ 5 × 10^−2^ at the beginning to be able to follow changes in both directions. This control experiment also supported the hypothesis that the enrichment we observed in the other mixes was olefins dependent. Moreover, we observed that when the wild type was mixed with the olefin-overproducing strain, an efficient enrichment of the latter strain in the upper phase of the culture was possible (mix C in Fig. 2a, ope:kan abundance changes from 20% to 100% after 20 h).

We further confirmed that such a simple sedimentation approach can indeed separate hydrocarbon-producing from non-producing cells of *M. luteus* by measuring the olefin content in hexane extracts, obtained from fractions of a mixture of the two strains ope and Δ*oleABCD*:kan (Fig. 2b). The two strains were grown separately to stationary phase in rich medium and equal amounts of cells were mixed (in a total volume of 800 ml) in a cylindrical glass vial and were let to stand undisturbed for 4 h. Samples were collected from the pure cultures, from the freshly mixed cell suspension (sample ‘1:1 Mix A’), and three samples from the mixed cell suspension after standing for 4 h, corresponding to the upper phase (Fr. 1), lower phase (Fr. 2) and the cell pellet, (Fr. P) as shown in Fig. 2b. The cells from all samples were collected by centrifugation and after normalizing by optical density were subjected to extraction with hexane. Quantitative GC–MS analysis of these samples clearly showed that the upper phase contained most of the olefins, which means most of the olefin-producing cells; while in the lower phase and the cell pellet, which formed after several hours of incubation, almost no olefins could be detected.

**Fig. 3:**
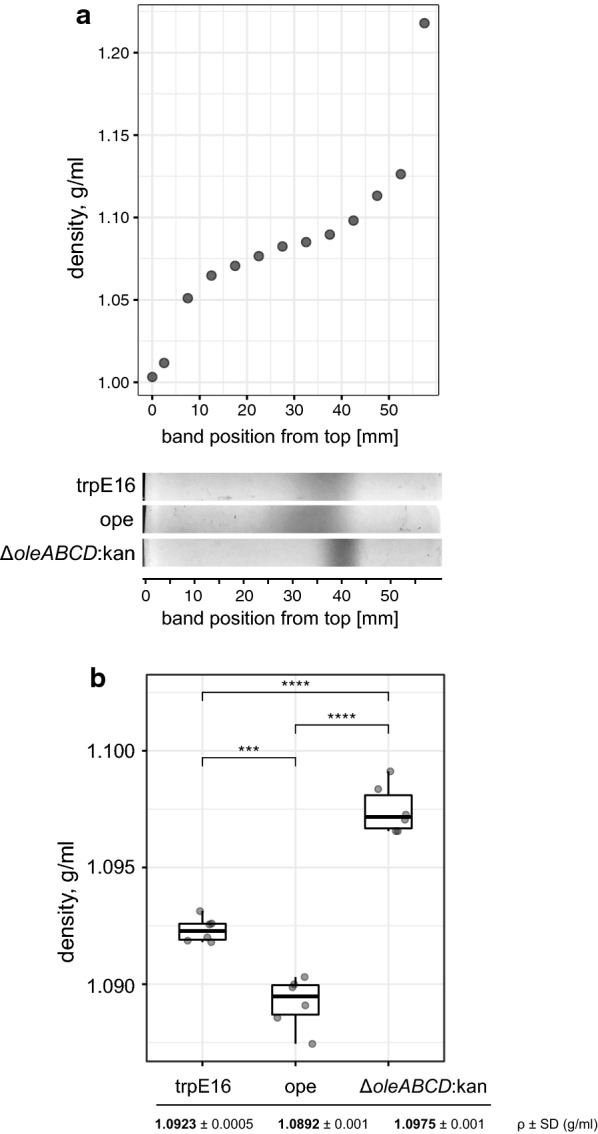
Cell density of the *M. luteus* strains trpE16, ope and Δ*oleABCD*:kan, determined by centrifugation to equilibrium in self-generated gradients of Percoll. **a** Top, density gradient of Percoll, formed by centrifugation of a 65% Percoll solution in 0.15 M NaCl (starting *ρ* = 1.09 g × ml^−1^) for 30 min at 23,000×*g* and 20 °C. The density was determined by measuring the refractive index with a digital refractometer of samples collected at the indicated positions, using a standard curve supplied by the manufacturer. Bottom, a representative result from the banding observed with cells of the indicated *M. luteus* strains, resuspended in 0.15 M NaCl and centrifuged at the above conditions. (B) Cell density of the *M. luteus* strains trpE16, ope and Δ*oleABCD*:kan (mean ± SD), determined by equilibrium centrifugation in self-generated Percoll gradients. The individual measurements from 6 centrifugations of cells from each strain are shown (*** significant at *p* ≤ 0.001, **** significant at *p* ≤ 0.0001, Welch’s unequal variances *t*-test)

Among several possible explanations of the observed phenotypic differences between olefin-producing and non-producing cells, the most obvious one is a difference in buoyant cell density. To test this, we performed buoyant density determination of the three *M. luteus* strains (trpE16, Δ*oleABCD*:kan and ope) by equilibrium centrifugation in Percoll gradients. Several independent cultures of each of the three strains were analyzed by centrifugation of the cells in in situ-formed Percoll gradients (initial density adjusted to 1.09 g × ml^−1^ in 0.15 M NaCl, centrifugation at 23,000×*g* for 30 min at 20 °C). A representative result from these measurements is shown in Fig. 3. Consistent with the observed differences in sedimentation, the buoyant densities of the examined strains showed small but well-detectable differences, the olefin-overproducing strain showing the lowest measured density. Indeed, it can be expected that the micrococcal olefins, which make up about 0.2% of the total dry cell weight in the wild type *M. luteus* strain ([13], and our own measurements), would have a measurable impact on the cell density. Considering an average density of 0.79 g × ml^−1^ for the olefins of *Micrococcus* (consisting mainly of C27 to C30 *mono*-alkenes), and a density of 1.0923 mg × ml^−1^ for the trpE16 strain, a fivefold increase in the cellular olefins (from 0.2 to 1% of the CDW as is the case in the ope strain, measured by GC–MS) would theoretically lead to a density of 1.0898 g × ml^−1^, which is in a remarkable agreement with the actually measured average density of the ope strain of 1.0892 g × ml^−1^ (Fig. 3).

Another interesting observation from the Percoll gradient centrifugation experiments was the broader density distribution of the cells of the olefin-producing strains in comparison to the cells of the olefin-deficient knockout strain, which can be seen as broader, less compact banding of the trpE16 and ope cells in Fig. 3a. A representation of the “compactness” of the cell banding for the three *Micrococcus* strains, obtained by plotting the optical density (which corresponds to cell density) of scanned images of the centrifuge tubes along the length of the tube, is shown in Additional file 1: Figure S1. This observation suggests a more heterogenic population (in terms of density) of cells in the olefin-producing strains compared to the deficient one. We are currently investigating if this density heterogeneity is caused by heterogeneity in the olefin content of the producer cells.

In the equilibrium density gradient centrifugation technique (isopycnic centrifugation), the cells are separated solely on the basis of differences in density, irrespective of size, which can play a role only before the equilibrium is reached [14]. The buoyant density differences between olefin-producing and non-producing cells which we observed must, therefore, be associated exclusively with the cell density, as the Percoll centrifugation experiments were performed to equilibrium. We corroborated this with microscopy observations and measurements of the average cell sizes of the three strains used in the Percoll experiments, e.g., E16, ope and Δ*oleABCD*:kan. No significant differences in the cell perimeter were observed for the three strains when they were grown to the stationary phase in rich medium (Additional file 2: Figure S2).

It is not clear if the small differences in density we measured by centrifugation in Percoll gradients are the sole reason for the pronounced phenotypic differences (rate of sedimentation) between olefin-producing and non-producing *Micrococcus* cells. Irrespective of the exact physical basis for the different behavior of olefin-producing and non-producing cells, the simple separation method described here can be used in various mutagenesis and enrichment protocols for isolating strains with increased olefin content. For example, it should be possible to enrich for olefin-overproducing mutants from a pool of randomly mutagenized *M. luteus* cells by repeated separation and outgrowth of the cells from the upper phase after settling. Also, it may be possible to accelerate the sedimentation rate by the “Boycott effect”, e.g., using inclined vessels [11].

It would be interesting to investigate if the olefin content-associated phenotypic differences which we describe for *Micrococcus* can be found and utilized also in other microorganisms with a natural or engineered hydrocarbon-producing capability.

## Conclusions

In summary, we show that cultures of *M. luteus* cells show distinct sedimentation behavior depending on the amounts of alkenes produced. Equilibrium centrifugation experiments in Percoll gradients of *M. luteus* mutants suggest that buoyant cell density is the primary cause for the observed differences in sedimentation behavior. We demonstrate how this phenotypic trait can be used to simply and efficiently fractionate *M. luteus* cells based on their hydrocarbon content. This approach can be useful in the isolation of olefin-overproducing mutants of *M. luteus* and probably other hydrocarbon-producing species.

## Methods

### Bacterial strains and growth conditions

A tryptophan auxotroph of the strain “*Micrococcus lysodeikticus*” (*M. luteus*) ISU, trpE16 [15, 16], was grown in LB medium at 30 °C and used as host for DNA manipulations and also as a reference wild type strain. Where appropriate, the growth media were supplemented with kanamycin (60 μg ml^−1^).

### Generation of *M. luteus* trpE16 mutants

The construction of the olefin-overproducing strain *M. luteus* ope is described in [17]. The olefin gene cluster deletion mutant Δ*oleABCD*:kan was generated by replacing the *ole* genes with a kanamycin resistance marker. DNA sequence regions (approx. 1 kb) upstream and downstream of the *ole* genes, as well as the kanamycin resistance marker were amplified by PCR, using Q5 polymerase (New England Biolabs). The PCR products with overlaps were assembled in vitro by Gibson Assembly (New England Biolabs). The in vitro Gibson assembly reactions (~ 0.2–0.4 μg DNA) were added directly to *M. luteus* cells for uptake via natural transformation and plated on LB plates supplemented with kanamycin. The correctness of the deletion in the genome was confirmed by PCR and sequencing.

### Percoll gradient centrifugation

A working solution of Percoll PLUS (Sigma Aldrich), with initial density of 1.130 g ml^−1^, was prepared by diluting Percoll to 72% in 0.15 M NaCl. Self-generating gradients of cells in Percoll were prepared by mixing 7.2 ml of Percoll working solution and 0.8 ml cell suspension in 0.15 m NaCl and centrifugation at 23,000×*g* at 20 °C for 30 min in a fixed-angle rotor (A27 8 × 50) in a Sorvall RC6+ centrifuge. The density of the self-generated gradients was determined by measuring the refractive index with a digital refractometer (ORD 1RS, Kern Optics, Germany) of control samples without cells, taken from the tubes at different distances from the bottom and using a standard curve (refractive index vs density) supplied by the manufacturer.

### Olefins extraction and GC–MS

The cells used for olefin extraction were collected by centrifugation in glass vials with PTFE screw-caps. The cell pellets were re-suspended in residual supernatant and 100 μl acetic acid was added and thoroughly mixed. One millilitre of methanol and 4 ml of hexane [amended with 10 μg ml^−1^ triacontane (Tokyo Chemical Industry) as an internal standard] were added. The whole mixture was shaken overnight at 640 rpm in a KS 130B shaker (IKA Werke, Germany). To facilitate phase separation, the samples were centrifuged shortly and the upper hexane phase was used for analysis. GC–MS analysis was performed using a Shimadzu QP2020 system, equipped with a Rxi-5 ms (30 m × 0.25 mm i.d. × 0.25 μm) capillary column. The samples (1 μl) were injected in split mode (split ratio 1:10) and helium flow was maintained at 1 ml min^−1^. The temperature program of the GC column was 40 °C for 3 min, ramp to 250 °C at 15 °C min^−1^, and 250 °C for 19 min. The MS was operated under ionization by electron impact at 70 eV and 200 °C; mass spectra were recorded at *m/z* range of 40–600. Quantification of the olefins by GC–MS was done by direct comparison of their TIC peak areas with the peak area of triacontane of known concentration, which had been added to the hexane used for extraction.

## Additional files

**Additional file 1: Figure S1.** Optical density (corresponding to cell density) plots after centrifugation of the *M. luteus* strains trpE16, ope and ΔoleABCD in self-generated Percoll density gradients. The cells from cultures grown to the stationary phase were washed in 0.15 M NaCl and centrifuged for 30 min at 23,000×*g* and 20 °C in Percoll with an initial density of 1.09 g × ml^−1^. The centrifuge tubes were scanned and the images were analyzed with ImageJ to produce the optical density plots.

**Additional file 2: Figure S2.** Histograms of cell perimeters of the *M. luteus* strains trpE16, ope and Δ*oleABCD*, grown to stationary phase in rich medium. The circumference of individual cells was measured on microscopic images (DIC) with ImageJ, using the Cell Magic Wand plugin (https://www.maxplanckflorida.org/fitzpatricklab/software/cellMagicWand/). The mean and standard deviation of the measurements are shown in the insets. The distributions of perimeters were not significantly different among the samples (Wilcoxon rank-sum test for all comparison pairs, p > 0.05).
