# Supplementary material for: Variances in cellular sedimentation behavior as an effective enrichment method of hydrocarbon-overproducing Micrococcus luteus strains
Source: Biotechnol Biofuels. 2018 Oct 20;11:288. doi: 10.1186/s13068-018-1286-6 (PMC6195688; doi:10.1186/s13068-018-1286-6)

Figure S1

Optical density (corresponding to cell density) plots after centrifugation of the *M. luteus* strains trpE16, ope and ΔoleABCD in self-generated Percoll density gradients. The cells from cultures grown to the stationary phase were washed in 0.15 M NaCl and centrifuged for 30 min at 23 000 *g* and 20°C in Percoll with an initial density of 1.09 g × ml^{-1}. The centrifuge tubes were scanned and the images were analysed with ImageJ to produce the optical density plots.

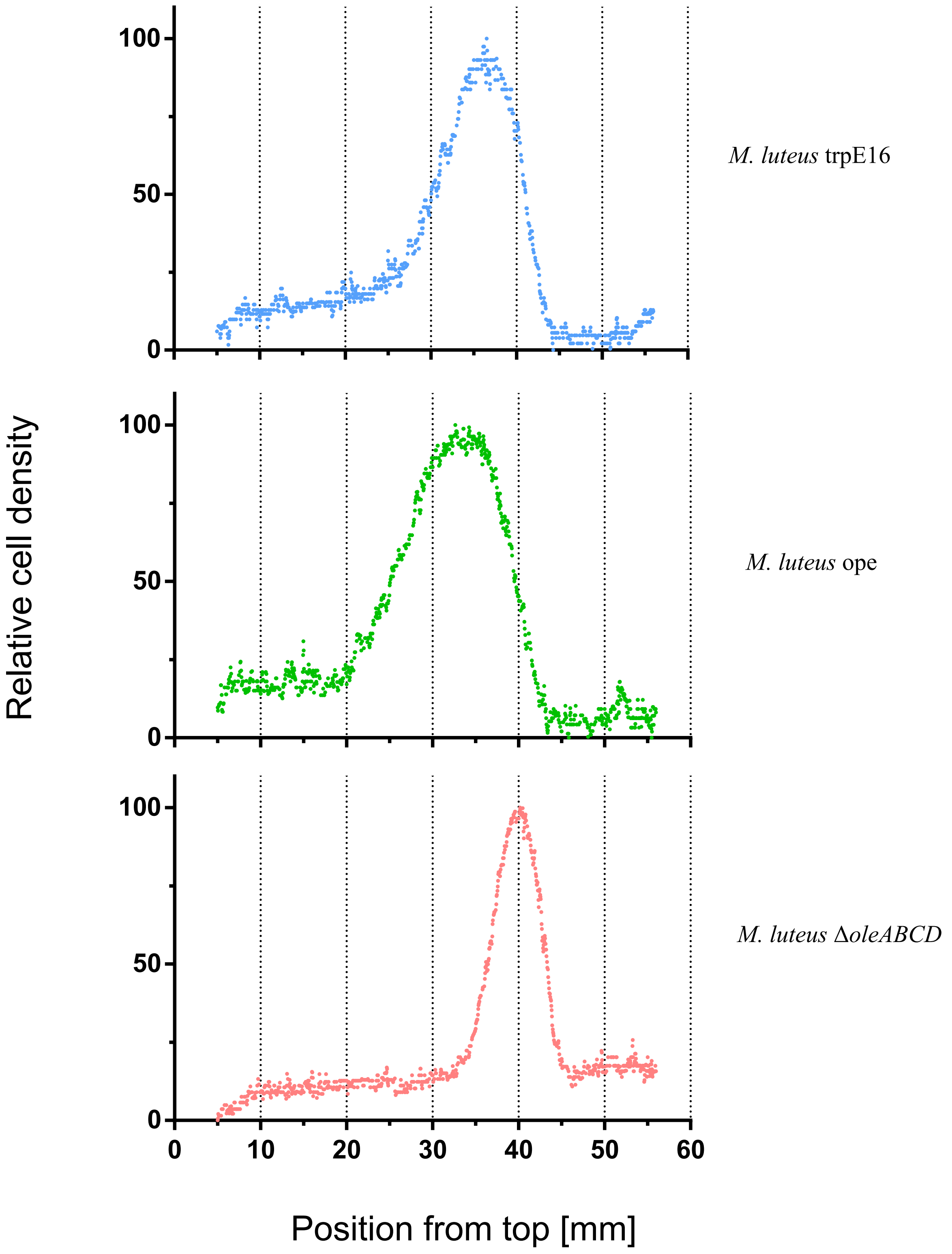

Supplement: Supplementary file 1 — Additional file 1: Figure S1. Optical density (corresponding to cell density) plots after centrifugation of the M. luteus strains trpE16, ope and ΔoleABCD in self-generated Percoll density gradients. The cells from cultures grown to the stationary phase were washed in 0.15 M NaCl and centrifuged for 30 min at 23,000×g and 20 °C in Percoll with an initial density of 1.09 g × ml−1. The centrifuge tubes were scanned and the images were analyzed with ImageJ to produce the optical density plots. [file 13068_2018_1286_MOESM1_ESM.pdf]
