# Supplementary material for: Variances in cellular sedimentation behavior as an effective enrichment method of hydrocarbon-overproducing Micrococcus luteus strains
Source: Biotechnol Biofuels. 2018 Oct 20;11:288. doi: 10.1186/s13068-018-1286-6 (PMC6195688; doi:10.1186/s13068-018-1286-6)

Figure S2

Histograms of cell perimeters of the *M. luteus* strains trpE16, ope and Δ*oleABCD*, grown to stationary phase in rich medium.
The circumference of individual cells were measured on microscopic images (DIC) with ImageJ, using the Cell Magic Wand plugin (https://www.maxplanckflorida.org/fitzpatricklab/software/cellMagicWand/).
The mean and standard deviations of the perimeters, as well as the number of measurements (n) are shown in the insets.
The distributions of cell perimeters were not significantly different among the three strains (Wilcoxon rank-sum test for all comparison pairs, P > 0.05).

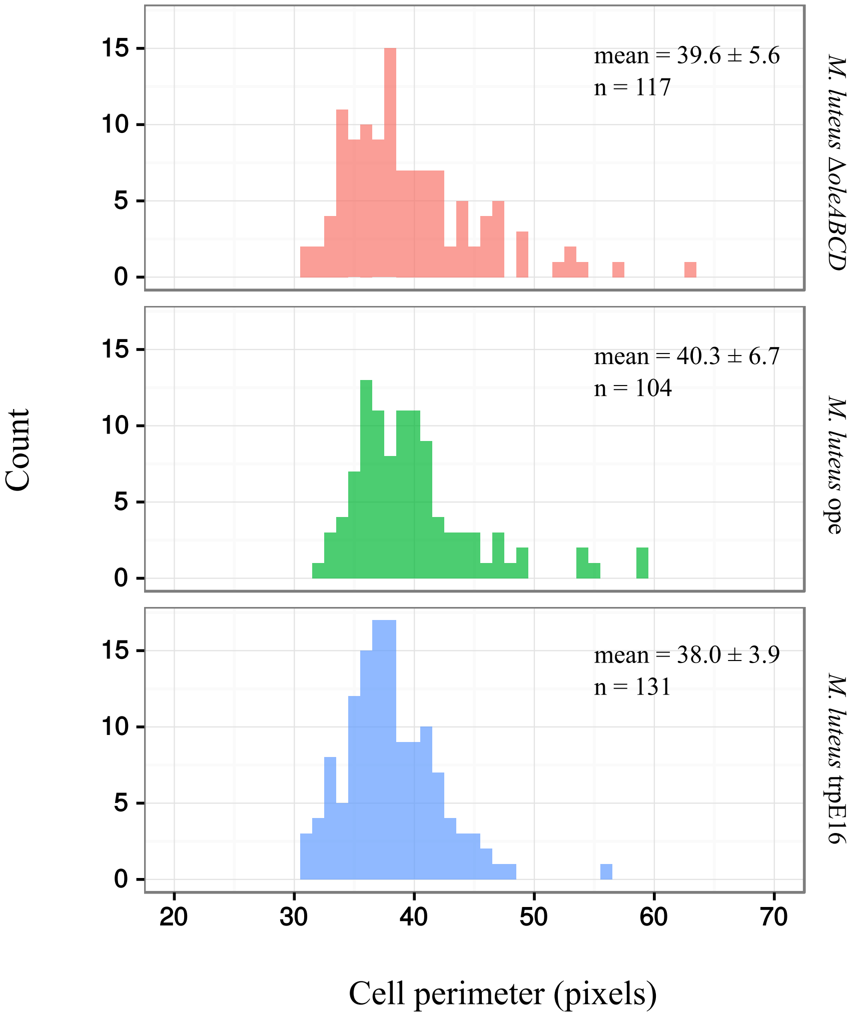

Supplement: Supplementary file 2 — Additional file 2: Figure S2. Histograms of cell perimeters of the M. luteus strains trpE16, ope and ΔoleABCD, grown to stationary phase in rich medium. The circumference of individual cells was measured on microscopic images (DIC) with ImageJ, using the Cell Magic Wand plugin (https://www.maxplanckflorida.org/fitzpatricklab/software/cellMagicWand/). The mean and standard deviation of the measurements are shown in the insets. The distributions of perimeters were not significantly different among the samples (Wilcoxon rank-sum test for all comparison pairs, p > 0.05). [file 13068_2018_1286_MOESM2_ESM.pdf]
